# SLC38A4 Amino Acid Transporter Expression Is Significantly Lower in Early Preterm Intrauterine Growth Restriction Complicated Placentas

**DOI:** 10.3390/ijms24010403

**Published:** 2022-12-26

**Authors:** Elif Kadife, Alesia Harper, Natasha De Alwis, Keegan Chien, Natalie Hannan, Fiona C. Brownfoot

**Affiliations:** 1 Obstetric Diagnostics and Therapeutics Group, Department of Obstetrics and Gynaecology, University of Melbourne, 163 Studley Road, Heidelberg, VIC 3084, Australia; 2 Mercy Perinatal, 163 Studley Road, Heidelberg, VIC 3084, Australia; 3 Therapeutics Discovery and Vascular Function Group, Department of Obstetrics and Gynaecology, University of Melbourne, 163 Studley Road, Heidelberg, VIC 3084, Australia

**Keywords:** fetal growth restriction, human, placenta, amino acid transporters, SLC38A1, SLC38A2, SLC38A4

## Abstract

Intrauterine growth restriction (IUGR), predominantly caused by placental insufficiency, affects partitioning of nutrients to the fetus. The system A sodium-coupled transporters (SNAT or SLC38), of types A1, A2, and A4, control non-essential amino acid uptake and supply. Here, we aimed to investigate the expression of these transporters across different placental disease cohorts and cells. To determine disease impact, transporter expressions at the gene (qPCR) and protein (western blots) level were assessed in gestationally matched placental tissues. Early (<34 weeks), and late (34–36 weeks) onset IUGR cases with/out preeclampsia were compared to preterm controls. We also investigated level of transporter expression in primary trophoblasts under glucose deprivation (n = 6) and hypoxia conditions (n = 7). SLC38A4 protein was significantly downregulated in early preterm pregnancies complicated with IUGR with/out preeclampsia. There were no differences in late preterm IUGR cohorts. Furthermore, we demonstrate for the first time in primary trophoblast cells, that gene expression of the transporters was sensitive to and induced by glucose starvation. SLC38A4 mRNA expression was also significantly upregulated in response to hypoxia. Thus, SLC38A4 expression was persistently low in early preterm IUGR pregnancies, regardless of disease aetiology. This suggests that gestational age at delivery, and consequently IUGR severity, may influence loss of its expression.

## 1. Introduction

Intrauterine growth restriction (IUGR) is a leading risk factor for stillbirth [1]. It is predominantly caused by placental insufficiency. Placental insufficiency, with reduced blood flow, also affects the transport and partitioning of nutrients across the maternal microvillous membranes to the fetal facing basal membranes of the syncytiotrophoblasts [2]. Nutrient deprivation further restricts the growth and healthy development of the fetus. Various factors control nutrient exchange across the placenta and, of these, system A transporters coordinate non-essential neutral amino acid uptake and supply [3]. System A transporters (SNAT or SLC38), types A1, A2, and A4, are sodium-dependent and pH-sensitive transporters. Of these, SLC38A1 and SLC38A2 are abundantly expressed in a range of organs, predominantly found in the heart and brain, while SLC38A4 was thought to be exclusively expressed by the liver. However, over the years, all SLC38 subtypes were found to be indispensable for the healthy development of the placenta and the fetus. Recently, SLC38A4 was found to be critical for placental development in mice, with the knockout leading to significant placental hypoplasia and a reduction in placental and fetal weights [4].

However, the expression and activity of these transporters in the dysfunctional placenta and related complications have not been fully established. In some human placentas, the level of reduction in system A transporter expression correlates with the severity of fetal growth restriction and the degree of fetal compromise [5,6]. In contrast, other studies involving animal models and in vitro trophoblast cultures propose enhanced expression and activity of these transporters to compensate for growth-restricted placentas [7,8,9]. However, compensation in response to maternal undernutrition appears to be specific to the mouse. In primates and rats, calorie, and protein restriction (respectively) cause a net decrease in transporter systems in the placenta, which precedes fetal growth restriction in these models [10,11]. In humans, different disease aetiologies and molecular factors also add further complexities to these findings. Uptake of amino acids is reduced in small for gestational age placentas, but not if they were complicated by preeclampsia [12,13]. Others show that transporter expression in the placenta is increased with fetal macrosomia cases, but not necessarily reduced with low birthweight. The discrepancy in findings may be because of the different clinical features and measures that define IUGR vs small for gestational age vs low birthweight, which can overlap and are sometimes used incorrectly or interchangeably [14,15]. Other limitations that exist in the current literature are the focus and study of one type of transporter, instead of all, as well as the use of inaccurately matched controls.

In this study, we have considered the expression of all three system A transporters, SLC38A1, SLC38A2, and SLC38A4. To address the impact of gestational age at delivery and disease severity, we separated our cohorts as early (>34 weeks) and late (34–36 weeks) preterm cases. To investigate the effect of IUGR and its different aetiologies, we considered cases with IUGR alone or combined with preeclampsia (PE), comparing them to appropriately matched gestational controls. To determine the impact of IUGR relevant stress conditions on expression, we isolated cytotrophoblast cells from term placentas and subjected these cells to glucose deprivation and hypoxia conditions in vitro.

## 2. Results

### 2.1. System A Sodium-Coupled Transporter Expression in Placentas from Pregnancies Complicated by Early Preterm IUGR and Preeclampsia

**Figure 1:**
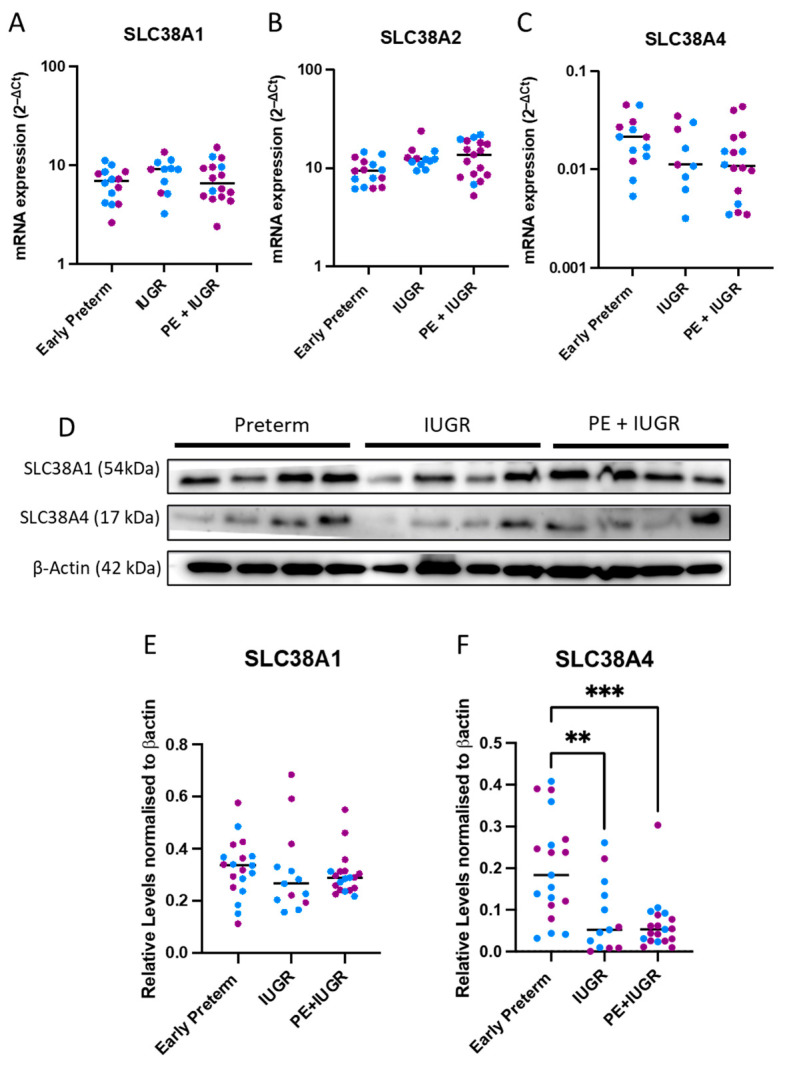
**In early preterm gestation patients, SLC38A4 protein levels were significantly reduced in IUGR complicated placentas.** In IUGR and PE + IUGR placentas, compared to preterm controls, mRNA expressions (assessed via qPCR) of *SLC38A1* (A), *SLC38A2* (B), and *SLC38A4* (C) were not different. Western blots (D) showed that, at the protein level, SLC38A1 (E) was unchanged between groups. SLC38A4 (F) was significantly decreased in both IUGR and PE + IUGR cohorts (n = 11–19 samples/cohort). Results are displayed as medians. ** *p* < 0.01, *** *p* < 0.001 (Kruskal Wallis non-parametric test for qPCR results and Western blot analysis). Blue dots (male pregnancies), purple dots (female pregnancies).

We assessed gene and protein expressions of SLC38A1, SLC38A2, and SLC38A4 in IUGR alone or preeclampsia + IUGR placental samples collected from early preterm (<34 weeks) gestation and compared these to gestationally matched uncomplicated preterm controls. In the early preterm cohort, there were no significant differences in *SLC38A1*, *SLC38A2,* and *SLC38A4* mRNA expression in the pathological placentas compared to the controls (Figure 1A–C).

To investigate whether gene expression directly correlates to protein levels, we conducted western blot analysis (Figure 1D). While there were no significant differences in SLC38A1 (Figure 1E), SLC38A4 protein (Figure 1F) expression was significantly lower in both IUGR cohorts compared to early preterm controls (*p* < 0.01 for IUGR and *p* < 0.001 for PE + IUGR). SLC38A2 proteins could not be reliably detected in these samples and were excluded from analysis in all experiments.

### 2.2. System A Sodium-Coupled Transporter Expression in Placentas from Pregnancies Complicated by Late Preterm IUGR and Preeclampsia

**Figure 2:**
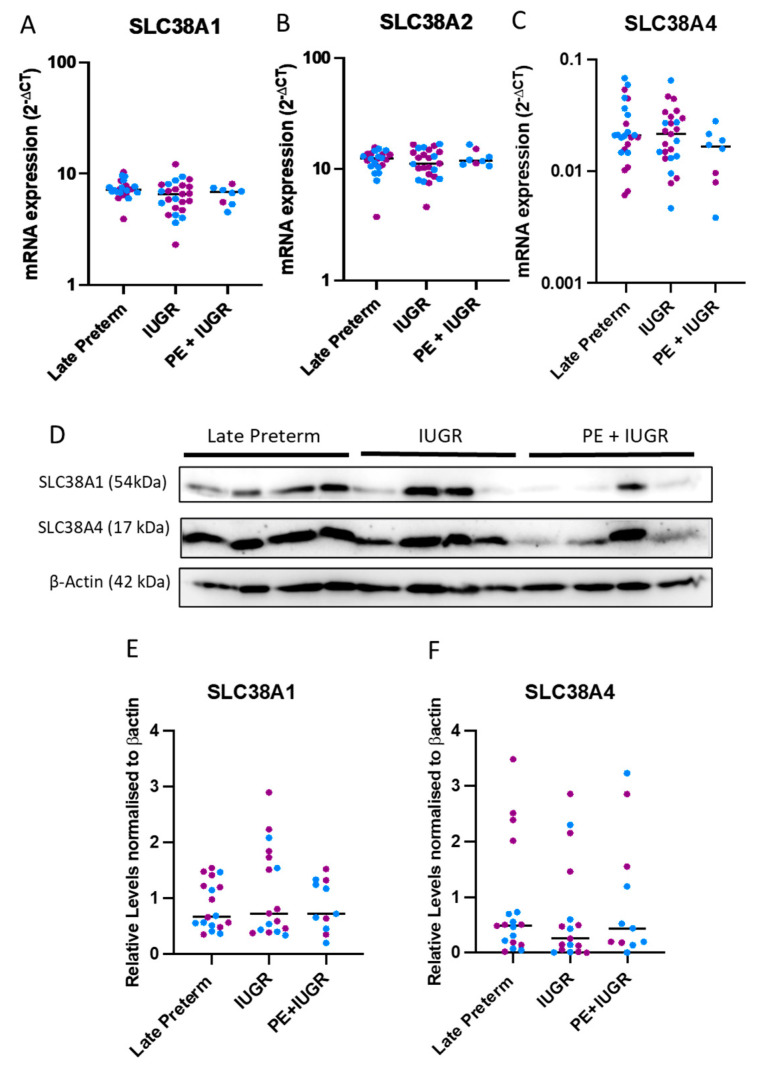
**In late gestation patients, the expression of transporters were indifferent between IUGR complicated placentas and preterm controls.** The mRNA levels (assessed via qPCR) of the transporters (**A**–**C**) and protein levels (assessed via Western blots) (**D**) when quantified (**E**,**F**) did not reveal changes to the expression of system A amino acid transporters. (n = 8–25 samples/cohort). Results are displayed as medians (Kruskal Wallis non-parametric test). Blue dots (male pregnancies), purple dots (female pregnancies).

We next assessed gene and protein expressions of SLC38A1, SLC38A2, and SLC38A4 in IUGR alone or in preeclampsia + IUGR placental samples collected from late preterm (34–36 weeks) gestation and compared these to gestationally matched controls. Like in early preterm tissues, gene expressions of the transporters were uniform across the late preterm cohorts (34–36 weeks average gestation) (Figure 2A–C). Western blot analysis (Figure 2D) revealed large variations at the protein level (Figure 2E,F). In contrast to the early preterm cases, the average protein expression of SLC38A1 and SLC38A4 in IUGR complicated placentas were not different than the controls (Figure 2E,F). These results highlight that gestational age may be a determining factor for transporter expression.

### 2.3. Expression of System A Sodium-Coupled Transporters in Trophoblast Cells under IUGR Relevant Stress Conditions

To determine the effect of IUGR relevant stress conditions on the expression of these transporters, we isolated trophoblasts from term placenta and exposed them to glucose deprivation and hypoxic conditions in vitro.

**Figure 3:**
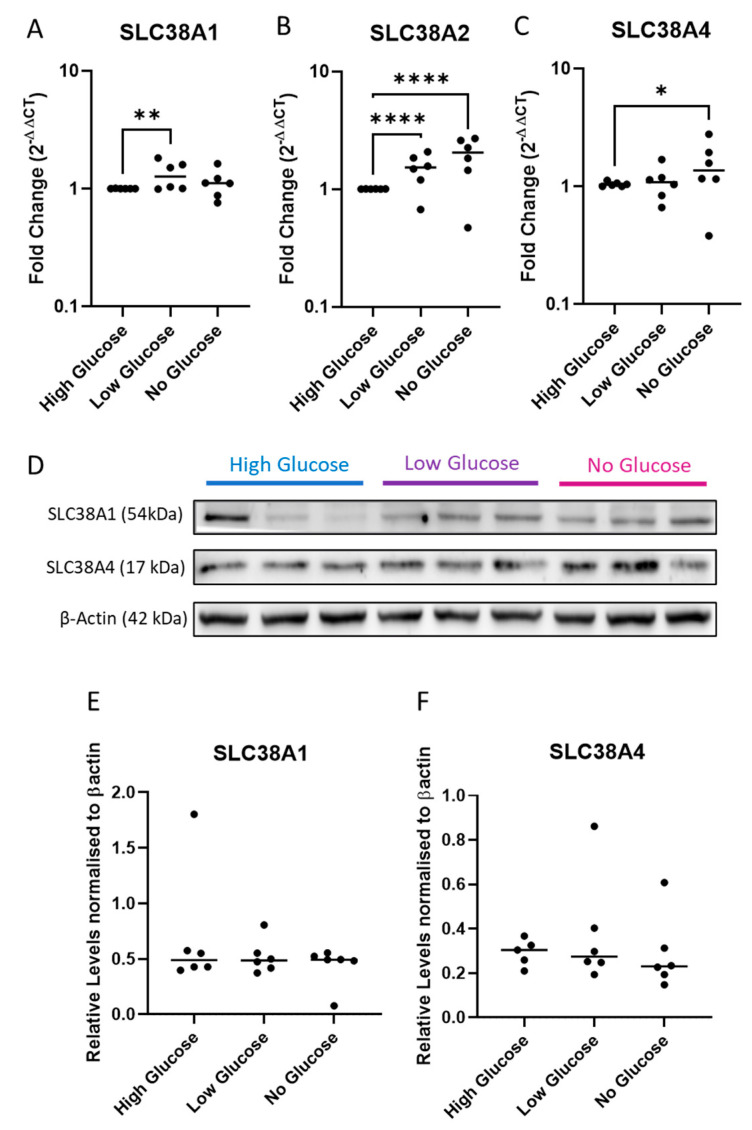
**Glucose deprivation significantly upregulated transporter expression in vitro trophoblast cultures.** Compared to normal media controls, glucose deprived cultures significantly upregulated *SLC38A1* (**A**), *SLC38A2* (**B**), and *SLC38A4* (**C**) expression (n = 6/condition). Additionally, in western blots (**D**), the expressions of SLC38A1 (**E**) and SLC38A4 (**F**) were not significantly altered (n = 6/condition). Results are displayed as medians. * *p* < 0.05, ** *p* < 0.01, **** *p* < 0.0001 (repeated measures are for one-way ANOVA for qPCR analysis and for Western blot analysis).

In the glucose deprivation experiments, cells were exposed to normal ‘high glucose’, ‘low glucose’, and ‘no glucose’ culture media conditions for 48 h. We demonstrate that glucose deprivation significantly alters gene expression of the transporters (Figure 3A–C). Of these, *SLC38A1* mRNA was significantly upregulated under low glucose conditions (*p* < 0.01), while complete starvation did not significantly alter its expression (Figure 3A). *SLC38A2* mRNA expression was induced in a stepwise manner in response to lowering glucose concentration (Figure 3B) (*p* < 0.001). Finally, *SLC38A4* mRNA expression was significantly induced in glucose starved cells (Figure 3C) (*p* < 0.05). In western blots (Figure 3D), the protein levels of SLC38A1 (Figure 3E) and SLC38A4 (Figure 3F) were unaltered.

**Figure 4:**
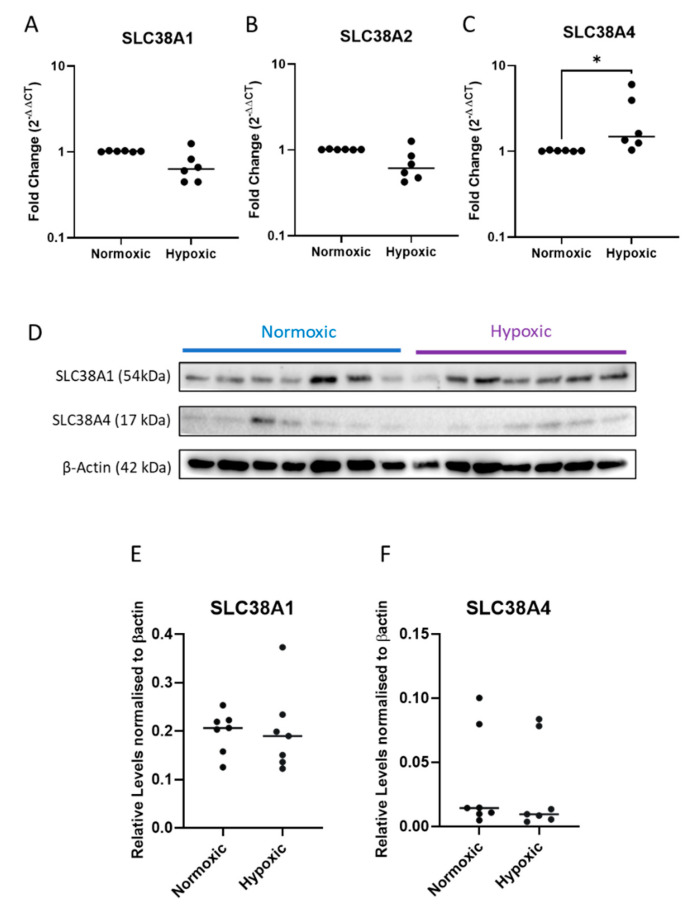
**IUGR relevant hypoxic stress conditions induced SLC38A4 expression at the gene level, however, protein expression remains unaffected in vitro trophoblast cultures.** Compared to normoxic controls, hypoxic trophoblasts showed insignificant changes to the expressions of *SLC38A1* (**A**) and *SLC38A2* (**B**). *SLC38A4* expression was significantly upregulated under hypoxic conditions (**C**) (n = 6/condition) (assessed via qPCR). Western blots (**D**) showed patient derived variations in response to hypoxia, which did not consistently alter the expressions of SLC38A1 (**E**) or SLC38A4 (**F**) (n = 7/condition). Results are displayed as medians * *p* < 0.05 (the paired *t*-test was used for qPCR analysis, and the Wilcoxon nonparametric test was used for Western blot analysis).

On average, hypoxic conditions (1% O_2_) tended to reduce *SLC38A1* (Figure 4A) and *SLC38A2* (Figure 4B) expression while significantly promoting *SLC38A4* expression (Figure 4C) (*p* < 0.05). Western blots (Figure 4D) showed similar expression of transporters between cells incubated under hypoxia and normoxia (8% O_2_) conditions in the 48-h experimental period (Figure 4E,F).

These results suggest that short-term changes in nutrient composition in media may be a more dominant factor affecting gene expression of these transporters than hypoxia. Nonetheless, *SLC38A4* expression was more sensitive to and significantly upregulated in response to glucose and oxygen deprivation stresses. However, gene changes do not reflect protein levels, which may be more tightly regulated or compensated in trophoblasts.

## 3. Discussion

Amino acid transport across the placenta and supply to the fetus are crucial for healthy development. System A sodium-coupled transporters are key to partitioning of neutral amino acids to the fetus. However, genetic targeting of these transporters has also revealed their role in supporting proliferation and cellular composition of the developing rodent placentas [4]. In human preeclampsia and IUGR, altered transporter expression and function reduces cord plasma amino acid concentrations and delivery to the fetus [6,16]. Yet, it is unclear whether the changes in transporters precede placental dysfunction and contribute to the condition or if they are impacted because of suboptimal cellular environments during pathogenesis [5,11]. To elucidate the influence of early vs. late disease onset, we investigated the expression of all three subtypes of system A transporters in matched gestation cohorts.

Our early preterm IUGR cohorts (<34 weeks) exhibited significant reduction in SLC38A4 protein expression compared to gestationally matched controls. Recently, SLC38A4 knockout in a mouse model caused placental hypoplasia and reduction in fetal weight [4]. Our investigation supports this finding, as the significant reduction in SLC38A4 appear to be an IUGR specific feature, regardless of comorbidities such as preeclampsia. Interestingly though, we show alternative regulation of gene and protein levels, as *SLC38A4* mRNA expression was not different than the early preterm controls. This type of disparity between gene and protein levels appears to be a feature of these transporters in healthy pregnancies as well. In normal placentas, SLC38A1 and SLC38A2 were found to be steady across first and third trimester samples. In contrast, while SLC38A4 mRNA significantly decreased, its protein expression increased across these gestations [17].

We did not observe any significant changes in transporter expression in the late gestation (>34 week) cohorts. Another study showed placentas from late rather than early onset IUGR had decreased SLC38A2 gene expression [18]. However, as acknowledged by the authors, there were small number of samples used for analysis in the early-onset cohort, and control samples were not appropriately matched for gestation. Furthermore, as highlighted by our findings, gene expression may not reflect protein or activity levels of these transporters. A strength of our study is the use of highly prized clinical samples from early and late preterm gestations, as well as appropriately matched controls. Therefore, gestational age, disease onset, and severity may be influencing factors for transporter expression.

Potential inter-species differences in transporter regulation have also been noted. In mice, studies suggest a compensatory mechanism where nutrient transporters are upregulated in the event of maternal undernutrition. While in non-human primates and rats, nutrient restriction causes a net decrease in transporter systems [10,11]. Reduced nutrient sensing and supply are important aspects of human IUGR [19,20]. Even though we did not take nutritional status into account when classifying our cohorts, there is existing evidence of amino acid deprivation positively regulating transporter expression in other systems [7,8]. However, little evidence exists about whether these transporters respond to other forms of nutritional inadequacies observed in IUGR. Low glucose transfer due to placental insufficiency causes premature activation of hepatic glucose production in IUGR fetuses [21]. This form of glucose is insulin resistant and predisposes the infants to metabolic disease. This has also been demonstrated in sheep models, where a state of prolonged fasting (>2 weeks) and hypoglycemia instigates growth restriction in sheep and alters glucose tolerance in the fetus [21]. In muscle cells, molecular regulators of glucose uptake concomitantly stimulate system A amino acid transport, crucial for protein synthesis [22,23]. Similarly, in rat livers, glucagon drives SLC38A2 expression [24,25]. To the best of our knowledge, the effect of glucose levels on transporter expression has not been previously reported in placenta cells. Here, we observe that glucose deprivation in primary trophoblasts significantly upregulates gene expression of all transporters, while the protein levels remain unchanged.

We went on to investigate transporter expression under another IUGR specific stress condition, hypoxia. Hypoxia induced HIF1α positively regulates SLC38A1 and SLC38A2 expression in adipose cells during obesity and in breast cancer cells, respectively [26,27]. In contrast to other systems, a placental study suggested that hypoxia reduced the expressions of a system A transporter in cytotrophoblast cultures in an oxygen level dependent manner and impeded amino acid uptake [28]. Our results showed that low oxygen significantly upregulated *SLC38A4* mRNA levels. While, *SLC38A1* and *SLC28A2* mRNA expression tended to be lower than the controls, this was not significant. Despite the significant changes to SLC38A4 protein in IUGR and preeclampsia + IUGR placentas, trophoblast hypoxia models did not alter the protein expressions of any of the transporters in vitro. This emphasizes the complexity of the human condition, which cannot be fully replicated in culture. A point of difference in findings between our study and those reported in cytotrophoblasts [28] may be that, by and large at the 48 h timepoint, our primary cytotrophoblasts fuse to form syncytiotrophoblasts. Our study is limited by sample number and the investigation of transporter expression, which may not be an accurate depiction of function. Hence, amino acid transport will be an important measure in determining biological consequences of stress conditions. This was shown in BeWo choriocarcinoma cell lines, where amino acid starvation had opposing effects on SLC38A1 and SLC38A2 expression, yet the overall activity of system A transporters was increased [29]. Therefore, short-lived stress may be compensated by adaptation through gene, protein, or functional upregulation, however, this may not be adequate in overcoming persistent pregnancy complications. Thus, even though the transporters serve a similar purpose of maintaining amino acid balance, they may be regulated by distinct molecular factors that can overlap depending on the cellular context.

## 4. Materials and Methods

### 4.1. Patient Samples

Placental tissues were collected with informed, written consent from patients at the University of Melbourne, Department of Obstetrics and Gynaecology at the Mercy Hospital for Women Ethics (#R11/34). Placental samples were collected from pregnancies complicated by severe early-onset preeclampsia and fetal growth restriction (requiring delivery ≤ 34 + 0 weeks gestation) at caesarean section. Fetal growth was defined as birthweight <10th centile, according to Australian population charts [30]. Preeclampsia was defined according to the American College of Obstetricians and Gynecologists guidelines [31]. Control preterm placental samples were collected from women with normotensive preterm pregnancies (≤ 34 + 0 weeks gestation) with fetal growth >10th birthweight centile at caesarean section. Preterm deliveries were predominantly for iatrogenic reasons other than fetal growth restriction (such as vasa previa) or premature rupture of membranes. Cases with evidence of chorioamnionitis (confirmed by placental histopathology) were excluded. At the time of collection, tissue samples were fixed in 4% paraformaldehyde and RNALater for 48 h, after which they were snap frozen and stored at −80 °C.

Early preterm (<34 weeks gestation) placental samples from cases of clinically diagnosed preeclampsia + intrauterine growth restriction (IUGR) (n = 22), IUGR alone (n = 13), and preterm controls (n = 20) were included in the analysis of genes and histological images. For late preterm (34–36 weeks), we utilised 24 preterm, 29 IUGR, and 11 preeclampsia + IUGR samples. All samples from the cohorts were included in the analysis and only excluded when there were experimental or technical errors within the experiments. Patient characteristics are presented in Table 1 and Table 2.

### 4.2. Isolating and Treating Primary Human Cytotrophoblast Cells and Placental Explants

Term placentas, from patients having elective caesarean sections, were sampled at four sites (clockwise direction), and the fetal and maternal membranes were removed. Then, the chorionic villi were used to isolate cytotrophoblast, as previously described [32]. Cells were seeded (5 × 10^5^ for RNA and 1 × 10^6^ for protein/well in technical triplicates/condition) and were incubated under 8% (normoxic) or 1% (hypoxic) O_2_ conditions at 37 °C, 5% CO_2_, or with DMEM (#11966025) culture media, supplemented with 1000 mg/L D-glucose of ‘high glucose’, 500mg/L of ‘low glucose’ and glucose-free media, or ‘no glucose’ conditions for 48 h under standard culture conditions (8% O_2_, 5% CO_2_, 37 °C). The RNA was collected for qPCR analysis (n = 6 for hypoxia/normoxia experiments and n = 6 for glucose deprivation) and protein for Western blot analysis (n = 7 for hypoxia/normoxia experiments and n = 6 for glucose deprivation). The samples for RNA and Westerns are not matched (from the same patients) for normoxia/hypoxia experiments, but are matched for glucose deprivation studies.

### 4.3. Quantitative Polymerase Chain Reaction (qPCR)

qPCR analysis was conducted on mRNA extracted from preterm control, preeclampsia + IUGR and IUGR placentas. Extraction of RNA from placental cytotrophoblasts and explants were performed with the RNAeasy mini kit (Qiagen, Valencia, CA, USA), according to the manufacturer’s instructions and quantified using the Nanodrop ND 1000 spectrophotometer (NanoDrop technologies Inc., Wilmington, DE, USA). RNA (0.2 μg) was converted to cDNA using the Applied Biosystems high-capacity cDNA reverse transcriptase kit (Life Technologies, Carlsbad, CA, USA) in line with the manufacturer’s guidelines. We assessed gene expressions of SLC38A1 (Hs01562175_m1), SLC38A2 (Hs01089954_m1), and SLC38A4 (Hs00394339_m1) (Taqman probes, Life Technologies) by real time PCR (RT-PCR) on the CFX 384 (Bio-Rad, Hercules, CA) using FAM-labeled Taqman universal PCR mastermix and its specific primer/probe set (Life Technologies) with the following run conditions: 50 °C for 2 min, 95 °C for 10 min, 95 °C for 15 s, and 60 °C for 1 min (40 cycles). Quantification was performed using the 2^–∆Ct^ method, normalising expression to the average expression of housekeeper genes CYC1 (Hs00357717_m1) and TOP1 (Hs00243257_m1). Results are presented as mRNA expression (2^–∆Ct^).

### 4.4. Western Blots

Protein was extracted in RIPA buffer and quantified using Pierce BCA kit (Thermo Fisher, Waltham, MA, USA), according to the manufacturer’s instructions. Protein (20 ug for patient samples and 7 µg for trophoblast) was loaded onto 12.5% gels and ran at 100 V before being transferred to a PVDF membrane at 100V for 1 h at 4 °C. The membranes were blocked in 5% skim milk and probed with primary antibodies SLC38A1 (NOVNBP259311, monoclonal) and SLC38A4 (NOVNBP155228, polyclonal) (Novus Biologicals) at 4 °C overnight. Secondary anti-rabbit-HRP antibodies were applied for 1 h RT, and then the membranes were imaged using chemiluminescence on the Bio-Rad ChemiDoc machine. The target bands were normalised against β-actin levels (#3700, cell signaling, Danvers, MA, US), and the band intensities were plotted a percentage change from normoxic controls for normoxia/hypoxia experiments and high glucose controls for glucose deprivation experiments.

### 4.5. Statistical Analysis

All experiments were performed with a minimum of three technical triplicates for each biological replicate, and there were at least three patients for each experiment. Statistical analysis was conducted using Kruskal-Wallis test for qPCR and Western blots of patient samples. Two-way ANOVA for glucose qPCR and Western blot experiments was used. Wilcoxon test for hypoxia qPCR and Western blots for hypoxia experiments were used. We used the GraphPad Prism 6 (GraphPad Software, La Jolla, CA, USA) for statistical analysis. All data were expressed as medians; *p* values < 0.05 were considered significant.

## 5. Conclusions

In conclusion, we have investigated gene and protein expressions of system A sodium-coupled transporters in both idiopathic IUGR and IUGR, with preeclampsia, to determine if disease aetiology has distinct impact on outcome. An important aspect of our study was also the categorisation of early (<34 weeks) and late (>34 weeks) preterm diseases and comparing these to appropriately matched preterm controls. As such, we demonstrated that early gestation cohort is marked by dysregulation of SLC38A4, which is significantly downregulated in the IUGR cohort. Furthermore, in exploring different forms of nutrient deprivations, we demonstrated, for the first time in primary trophoblast cells, that the gene expression of the transporters is sensitive to and is upregulated by glucose starvation. Moreover, changes to expression and sensitivities to cellular stresses are not uniform between transporter subtypes. There are likely different factors influencing the expression and function of these transporters in placental pathologies.

## Figures and Tables

**Table 1:** Clinical characteristics of <34 week patient cohort.

Patient Characteristics	Preterm (n = 20)	IUGR (n = 13)	IUGR + PE (n = 22)
Maternal age (years)	32 (25–37)	30.5 (24.3–36)	31 (25–32)
Body–mass index (kg/m^2^)	29.2 (25.3–35.2)	23.6 (18.6–30.5)	28 (25–35.5)
Smoking during pregnancy (%)	4 (27%)	5 (42%)	1 (5.9%)
Diabetes during pregnancy (%)	3 (21%)	3 (25%)	1 (5.9%)
Gestational age at delivery (weeks)	30 (29–32)	31.5 (30.1–32.8)	30.1 (26.9–31.3)
Birthweight (grams)	1585 (1237–2000)	993.5 (861–1257.3)	911 (557.5–1173)
Fetal Sex (Female/Male)	9/11	8/5	14/8

**Table 2:** Clinical characteristics of >34 Week Patient Cohort.

Patient Characteristics	Preterm (n = 24)	IUGR (n = 25)	IUGR + PE (n = 11)
Maternal age (years)	30.5 (28–34.8)	32.24 (30–35.5)	35.5 (27.3–36)
Body-mass index (kg/m^2^)	24 (21–27)	26.05 (22.2–28)	23.5 (20.5–25.7)
Smoking during pregnancy (%)	1 (4.2%)	2 (6.9%)	0 (0%)
Diabetes during pregnancy (%)	2 (8.3%)	6 (21%)	3 (38%)
Gestational age at delivery, weeks	34.9 (34.6–35.9)	35.52 (34.4–37.4)	36.1 (34.5–36.3)
Birthweight, grams	2605 (2239.8–2810)	1932.68 (1672.5–2240)	1958 (1774.5–2085)
Fetal Sex (Female/Male)	11/13	16/9	4/7

## Data Availability

The data presented in this study are available on request from the corresponding author.
